# Reducing intervention- and research-induced inequalities to tackle the digital divide in health promotion

**DOI:** 10.1186/s12939-023-02055-6

**Published:** 2023-12-04

**Authors:** Laura M. König, Rebecca A. Krukowski, Emmanuel Kuntsche, Heide Busse, Laura Gumbert, Kathrin Gemesi, Efrat Neter, Nor Firdous Mohamed, Kathryn M. Ross, Yetunde O. John-Akinola, Rosie Cooper, Anila Allmeta, Anabelle Macedo Silva, Cynthia C. Forbes, Max J. Western

**Affiliations:** 1 Faculty of Life Sciences: Food, Nutrition and Health, University of Bayreuth, Bayreuth, Germany; 2 Faculty of Psychology, University of Vienna, Wächtergasse 1/504, 1010 Vienna, Austria; 3 Department of Public Health Sciences, School of Medicine, University of Virginia, Charlottesville, USA; 4 Centre for Alcohol Policy Research, La Trobe University, Melbourne, Australia; 5 Leibniz Institute for Prevention Research and Epidemiology - BIPS, Bremen, Germany; 6 Leibniz Science Campus Digital Public Health Bremen, Bremen, Germany; 7 SMA Europe E.V, Freiburg, Germany; 8 Institute for Nutritional Medicine, School of Medicine and Health, Technical University of Munich, Munich, Germany; 9 Ruppin Academic Center, Emek Hefer, Israel; 10 Department of Psychology, Faculty of Human Development, University Pendidikan Sultan Idris, Tanjong Malim, Malaysia; 11 University of Florida, Gainesville, USA; 12 Department of Health Promotion and Education, Faculty of Public Health, College of Medicine, University of Ibadan, Ibadan, Nigeria; 13 Nuffield Department of Primary Care Health Sciences, University of Oxford, Oxford, UK; 14 Instituto de Saúde Coletiva, Universidade Federal do Rio de Janeiro, Rio de Janeiro, Brazil; 15 Wolfson Palliative Care Research Centre, Hull York Medical School, University of Hull, Hull, UK; 16 Centre for Motivation and Health Behaviour Change, Department for Health, University of Bath, Bath, UK

**Keywords:** Digital technology, Health inequalities, Health inequity, Internet-based intervention, Determinants of health, Public health, Global health

## Abstract

Social inequalities are an important contributor to the global burden of disease within and between countries. Using digital technology in health promotion and healthcare is seen by some as a potential lever to reduce these inequalities; however, research suggests that digital technology risks re-enacting or evening widening disparities. Most research on this digital health divide focuses on a small number of social inequality indicators and stems from Western, educated, industrialized, rich, and democratic (WEIRD) countries. There is a need for systematic, international, and interdisciplinary contextualized research on the impact of social inequality indicators in digital health as well as the underlying mechanisms of this digital divide across the globe to reduce health disparities. In June 2023, eighteen multi-disciplinary researchers representing thirteen countries from six continents came together to discuss current issues in the field of digital health promotion and healthcare contributing to the digital divide. Ways that current practices in research contribute to the digital health divide were explored, including intervention development, testing, and implementation. Based on the dialogue, we provide suggestions for overcoming barriers and improving practices across disciplines, countries, and sectors. The research community must actively advocate for system-level changes regarding policy and research to reduce the digital divide and so improve digital health for all.

Social inequalities are an important determinant of the global burden of disease within and between countries; this is true for both high and low- and middle-income countries [29]. Disparities in health-related behaviors, health outcomes, and healthcare access may occur based on a range of individual characteristics such as gender, age, disability, education, income, race, occupation, or urban vs rural residency [17, 21, 31]. For instance, research conducted in high income countries suggests that those with a low socioeconomic status (SES) are less likely to engage in sufficient amounts of physical activity [26]. Similarly, rates of cigarette smoking are three times greater among individuals with lower incomes compared to those with higher incomes within the United States [5]. Disparities may also occur due to structural and social causes, such as divides in education or housing, which may put certain populations at risk of not seeking or receiving appropriate care [31]. Accordingly, disparities may also exist between geographical regions. For instance, the highest rates of diet-related deaths have been observed in low- and middle-income countries, while lowest rates were observed in high-income countries [1]. Reducing social inequality would have a massive impact on population health, preventing countless premature deaths and increasing global quality of life. Accordingly, reducing social inequalities is at the core of the United Nations Sustainability Goals, e.g., goal 6 (gender equality) and goal 10 (reduced inequalities).

Digital health, that is, the use of digital technology in health promotion and healthcare and related areas, has been proposed as an important vehicle to reduce disparities in health promotion and healthcare. Stakeholders assume that given the high and rising penetration, by using technology, access to high quality care may be improved at low cost [28]. However, social inequality is often associated with lower use of digital technology, resulting in a digital health divide [12]. For example, older adults are less likely to use the internet [20] and smartphones [23]. There is also a digital divide between countries, with a larger share of people in the Global North having access to the internet than in the Global South. Patterns of social inequality regarding internet access, however, are similar between countries. For instance, people with lower incomes are less likely to have access to the internet in both regions [11]. Given that the use of digital health technology often requires access to the internet and/or a computer or smartphone, these access disparities challenge the assumption that digital health technology is making health promotion and healthcare more equitable.

This notion of a digital divide is furthermore supported by recent reviews on digital health technology use and effectiveness [16]. For instance, young people with a high level of education and higher income are more likely to use digital health technology [19]. Furthermore, high SES populations benefit from digital interventions for physical activity, while low SES groups do not [30]. Thus, there is the risk that digital health technologies will not only re-enact, but also widen health disparities, but research on testing whether this occurs and how it may be prevented is sparse. Western et al. [30] only identified 19 randomized-controlled trials that specifically examined effectiveness for improving physical activity behavior based on SES. Furthermore, the range of social inequality indicators studied is narrow. Szinay et al. [27] synthesized the literature on a potential digital divide in the uptake of, engagement with, and efficacy of exclusively mobile interventions for weight-related behaviors and found that out of the 16 publications included, the majority focused on age, gender, education and ethnicity/race. Other inequality indicators included in the PROGRESS-plus framework [21] such as income, occupation, urban vs. rural residency, or sexual orientation have not received similar levels of attention to date. Finally, digital health research is conducted almost exclusively in Western, educated, industrialized, rich, and democratic (WEIRD) countries, with the majority of studies stemming from North America, Europe, and Australia and New Zealand [27, 30]. There is thus a need for a systematic, international, and interdisciplinary study of the impact of social inequality indicators in digital health as well as the underlying mechanisms of this digital divide across the globe to reduce health disparities.

Exchange between communities from different continents, e.g., at conferences, is rare due to costs and burden of international travel; in-person exchange has been further complicated by the COVID-19 pandemic. In addition, different research traditions emphasize different social inequality indicators. For example, while studying disparities based on race is common in the United States, the topic is usually not addressed in Germany for historic reasons.

To advance the study of a digital divide in health promotion both within and between countries, international collaboration is vital. For this reason, we conducted an international expert workshop that was held in Kulmbach, Germany, from 20 to 23 June 2023. Eighteen researchers represented eight countries (Australia, Brazil, Germany, Israel, Malaysia, Nigeria, United Kingdom, United States of America) through their current residency and contributed perspectives from an additional five due to their countries of origin (Albania, Canada, Lebanon, Mexico, Spain). Their research backgrounds were in psychology, behavioral science, public health and health promotion, nutritional science, law, addiction science, physical activity promotion, and health economics. Through a series of presentations and group discussions, workshop participants synthesized the current state of research, discussed current issues in the field, and formed an international network on the study of the digital divide in health promotion.

In the following, we present the results of these discussions, focusing on the main challenges in the field of digital health promotion and healthcare regarding social inequality, to stimulate further research in this area and ultimately improve (digital) health for all. First, we present issues related to intervention-induced inequalities (i.e., inequalities that arise through the use of digital technology in health promotion and healthcare). In this section, issues are clustered following the digital rainbow model [12]. This model is based on the Whitehead and Dahlgren [31] framework, which organizes social determinants of health (and subsequent interventions to reduce disparities) in five hierarchical levels, ranging in descending order from general socio-economic, cultural, and environmental conditions to stable individual-level factors such as age and gender. We use this model to highlight the different levels on which the digital divide may operate. Afterwards, we present issues related to research-induced inequalities, where we reflect on the contribution of digital health research to the digital divide, and how these may be overcome through changes in research practices.

## Intervention-induced inequalities

### Socio-economic, cultural, and environmental conditions

These general conditions may influence population health through providing opportunities and removing barriers, e.g., through legislation. We know from non-digital interventions that interventions operating at higher levels (e.g., smoking ban and taxation policy) of the system are more likely to impact health at scale [6]. Thus, systems-level approaches may also be required to address the digital divide. For instance, digital (health) literacy can be increased through school-based interventions, exposing students to digital technology, and teaching them how to use it sensibly to achieve their goals such as searching for trustworthy health-related information and changing health behaviors (see e.g., [10] for an example). However, these approaches require the readiness of the political system to support these endeavors, e.g., through providing adequate funding and making the relevant training mandatory for teachers; not all political systems might be ready to tackle these inequalities [12]. Furthermore, regulatory standards for digital health tools are needed to ensure that they are fit for purpose. In some countries such as Germany, regulatory frameworks exist that state how digital health tools need to be evaluated in order for them to become recognized treatment options, including the opportunity for costs to be covered by health insurances [25]. In this way, high quality digital health tools can be offered to patients independently of their SES. To date, however, evaluation criteria do not take into account whether these trials included marginalized groups or had diverse samples. It is thus to be expected that efficacy is only assessed for certain subgroups of the population which may benefit more from digital interventions [24, 30]. Similarly, global initiatives such as ‘The Global Digital Health Partnership’^1^ do not include diversity in their assessment. To counteract this trend, stricter evaluation criteria regarding inclusivity and diversity of samples are needed, which again could be implemented through legislation.

### Living and working conditions

Living and working conditions also influence digital health inequalities. For instance, certain populations do not have access to digital technology and are thus considered digitally poor. Especially in Global South countries such as Nigeria, internet access is often limited due to restricted availability and affordability through “pay as you go” service plans. Socioeconomically deprived populations may thus not be able to afford internet access or may only use it sparsely to keep costs low. As a consequence, not all settings and contexts are suitable for digital health interventions, or the specific modality needs to be reconsidered (e.g., use text messaging instead of a website, or app features that can be downloaded when a user has access to WiFi, without requiring consistent access to an internet signal); this fit needs to be considered when digital tools are suggested or implemented as a public health measure.

### Social and community networks

Social and community networks may act as important guides to digital resources. For instance, individuals interested in using digital health technology may ask the advice of healthcare professionals (including general practitioners, nurses, dietitians, and fitness coaches) regarding whether and which tool to use [13]. Several barriers, such as low acceptance, lack of competencies, lack of awareness of digital health tools and the potential benefits, or preconceived ideas and stereotypes of whom these tools are suited for among providers may influence whether and to whom digital health tools are recommended and so discourage the use of digital health tools among the intended users [7]. Healthcare professionals, including the clinical workforce, thus should be involved in the development of digital health tools to ensure that they fit their requirements. In addition, campaigns are needed to promote awareness, provide training on how to use digital health tools to complement care, and tackle stereotypes about digital health tool users (e.g., older adults not being capable of handling technology) or privacy concerns to boost uptake. Moreover, conflicts related to cultural, moral, and religious backgrounds among users can hinder the acceptance of digital health solutions, especially in countries with various cultural influences and resulting differences in individualistic versus collectivistic tendencies [7], such as Malaysia. This may impact both engagement with a tool, with tools not fitting one’s cultural norms and expectations being abandoned [13], as well as interactions between healthcare professionals and patients when discussing the use of digital health tools. Culturally sensitive digital health tools are thus needed, which again highlights the importance of expanding digital health development and testing beyond WEIRD countries.

### Individual and lifestyle factors

Also individual and lifestyle factors play an important role in engaging in healthy and unhealthy behaviors; in the context of digital interventions, however, little is known to date about mechanisms underlying the uptake of, engagement with, and effectiveness of digital health tools by different individual factors (e.g., SES, age, gender). Understanding these mechanisms, be it motivation, familiarity and technology skill and literacy developed in another domain such as work, attitudes, social norms, or other personal factors, would allow the transfer of knowledge from one digital tool to another. Gaining this knowledge requires a common understanding of social inequality indicators and standardized measurement, which is especially difficult for SES, given the variability of median income and education levels across countries [30]. Developing an understanding of the personal influences of digital health access and benefits also requires a more concerted effort to engage those disadvantaged in research studies, something our scientific fields have collectively struggled to achieve.

## Research-induced inequalities

### Study planning, design, and analysis

Throughout the research process, from study conception, through recruitment to study reporting, researchers often make decisions (e.g., about eligibility criteria, recruitment strategies) unconsciously that can impact the applicability of the study to marginalized groups and thus limit generalizability of the findings. First and foremost, researchers need to be made aware of the potential impact of their decisions and provided with solutions for how to improve their studies to not increase (and potentially even reduce) health disparities. This principle applies both to tackling social inequalities in general and to digital health inequalities specifically. In line with contemporary changes to research practices in the behavioral sciences, changes need to be introduced to existing templates for study registration (e.g., in clinical trial registries or preregistration websites such as the Open Science Framework [OSF] or aspredicted.org) to include a wider range of potential inequality indicators (see e.g., Cochrane’s PROGRESS Plus framework, [21]). Similarly, to aid transparency around addressing inequalities, existing reporting guidelines such as CONSORT or STROBE should be extended to include social inequality indicators in baseline assessments and for potential differences to be explicitly checked when analyzing the data, e.g., through sensitivity analyses [8]. Since many journals require reporting checklists to be included upon submission, and preregistration of studies is becoming more common (even for study designs other than clinical trials), it is likely that these structural changes will lead to a relatively quick change in research practices [14]. Finally, researchers should involve key stakeholders, including patients, clinicians, community members, and potential end-users of digital health interventions, in the study planning process as early as possible (e.g., through community-based participatory research or patient and public involvement [4], to address research questions that are relevant and important to them and to design study materials in accordance with their needs and expectations.

### Recruitment

When recruiting participants for any study, it is important to take into account who is using which channels, and thus who would be most likely to engage with study advertisements; this is especially important when using digital recruitment methods. Different recruitment modalities reach different population subgroups [22], and research should be conducted with an understanding of the recruitment strategies and modalities that are more likely to reach certain population groups. In addition, recruitment benchmarks of specific population groups should be established, so that researchers can periodically compare actual recruitment proportions to the established benchmarks and know when they may need to pivot to new recruitment strategies/modalities (i.e., if the recruited sample is becoming too homogenous). Decisions about how and where to recruit can then be made deliberately to reach the targeted group or to achieve desired sample diversity. Similarly, inequalities may occur in all stages of the research process; this also includes enrollment and engagement, and not only intervention effectiveness. All stages of the research process thus need to be critically examined regarding potential harmful side effects that may widen inequalities.

### Local vs global scope

The digital health divide is a global issue; research on this topic thus should not be limited to individual countries. Furthermore, the issue is interdisciplinary in nature, as highlighted by the socioecological models used to describe the contributing factors [12] as well as the fact that contributions of several disciplines including computer science or engineering as well as psychology, sociology, and other behavioral sciences are needed to successfully develop digital health interventions. Finally, intersectoral collaboration, that is between research, clinical practice, and industry, is urgently needed to ensure that the developed tools are fit for purpose, widely implemented and tested, appropriately regulated, and maintained in line with the technology infrastructure of all [2]. For this to be achieved, new funding structures need to be implemented that are available to larger, international (cross-continent) research consortia, and that also appropriately address the needs of interdisciplinary (i.e., more time for discussions and planning, discussion of relevant research outputs) and intersectoral collaboration (i.e. more rapid development cycles in industry). Furthermore, international collaborations allow for studies to be conducted in the participants’ local language to ensure that English language skills are no barrier to participation. Yet, these projects are rare and typically require funding by multinational organizations (e.g., the European Union, which is still largely limited to EU countries). This issue may be overcome through big team science such as Many Labs projects (see [14], for a discussion), which typically involve hundreds of researchers from various countries who pool their resources (personnel and study funding) to collect data using the same (translated) measures, and potentially also conduct experiments or interventions using the same (translated) materials. In this vein, effects may be compared between countries and cultures to see whether effects are indeed generalizable.

### Data collection methods

Furthermore, improvements are required regarding research methods used, since these methods might impact the willingness of individuals to take part in a study. For example, the completion of lengthy questionnaires requires the availability of time and ability to understand the questions and response options, which may be limited in populations with low literacy. Importantly, research also suggests that response scales are interpreted differently depending on study participants’ cultural background [9]. It is thus advised that researchers think critically about the research methods used and that these measures are appropriately adapted to the target group, for example by making materials culturally appropriate or adjusting sampling and data collection methods to preferences and use habits (e.g., digital vs. pen-and-paper, computer vs. smartphone). Amongst others, this could be achieved by involving participant representatives early in the research process through co-creation. Similarly, inequalities may be exacerbated by the time commitment required to take part in a study, especially if study participation requires visits to a study center, which are usually located in the center of larger towns or cities. Accordingly, rural populations or those living in more affordable but less central housing may experience difficulties in reaching these centers; this difference may be exacerbated for low SES populations who might not own a car or cannot afford parking in city centers. While these issues are relevant to any (health-related) study, digital tools might provide a (partial) solution to the issue: Remote assessments (e.g., via telephone or video calls, text messages) may bridge this gap and increase reach [18]. Finally, study participation should be appropriately incentivized; this may be especially important when aiming to recruit disadvantaged populations who may need to choose between taking part in a study and using the time to generate income [3].

### Access to research results

Finally, research results should be made openly accessible and summarized to facilitate knowledge exchange among researchers. For instance, living reviews, i.e., databases that are updated at regular intervals or even automatically, can provide an up-to-date overview of findings. Similar databases containing developed and tested digital interventions, including information on whether they worked, for whom, and under which conditions, would accelerate research and prevent research waste [15]. By making these results open access, the existing digital divide in academic publishing due to high costs of journal subscriptions would be simultaneously tackled, which would allow researchers from low- and middle-income countries to participate more equally in academic collaborations. Publishing in open access journals, however, is expensive; especially researchers in low- and middle-income countries may not be able to afford the publishing fees. Fee waivers—or diamond open access models that rely on sponsorships by organizations rather than payments by individual research teams—are thus essential to promote knowledge exchange. In addition, authors might want to consider self-archiving manuscripts (known as “green open access”) in repositories such as OSF or other preprint servers; these services are usually available free of charge. International collaborations would also be beneficial to overcoming language barriers, since not all study reports are published in English; by involving an international research team, publications in a wider range of languages could be reviewed, and results could be made accessible to a wider audience through translations. Emerging artificial intelligence technologies could be used to help with translations. Furthermore, through science communication activities (e.g., on social media), researchers may translate their findings to key stakeholders (e.g., clinicians) or lay people, thereby acting not only as a translator between audiences, but also removing barriers related to health literacy (e.g., by using clear language and avoiding jargon).

## Conclusions

Without careful consideration of the points discussed in this article, the introduction of digital tools in health promotion and healthcare risks widening rather than reducing existing health inequalities. The digital divide is fueled by a range of intervention-induced inequalities, relating to the way digital interventions are currently tested and distributed, and research-induced inequalities, relating to the way digital health interventions are developed and evaluated. Changes in both areas are urgently needed to address the digital health divide; this applies to a wide range of scientific disciplines that are typically involved in digital health intervention development and testing, as well as industry and the public sector. Ultimately, to improve practices resulting in or widening the digital health divide, changes in political and research systems are urgently needed, and the research community has to actively advocate for improving (digital) health for all.

## Data Availability

Not applicable.

## Footnotes

1 https://www.healthit.gov/topic/global-digital-health-partnership#:~:text=The%20Global%20Digital%20Health%20Partnership%20is%20a%20collaboration%20of%2030,and%20advance%20mutually%20beneficial%20projects

## References

[1] Afshin A, Sur PJ, Fay KA, Cornaby L, Ferrara G, Salama JS, Murray CJ (2019). Health effects of dietary risks in 195 countries, 1990–2017: a systematic analysis for the Global Burden of Disease Study 2017. Lancet.

[2] Arigo D, Jake-Schoffman DE, Wolin K, Beckjord E, Hekler EB, Pagoto SL (2019). The history and future of digital health in the field of behavioral medicine. J Behav Med.

[3] Bierer BE, White SA, Gelinas L, Strauss DH (2021). Fair payment and just benefits to enhance diversity in clinical research. J Clin Transl Sci.

[4] Cargo M, Mercer SL (2008). The value and challenges of participatory research: strengthening its practice. Annu Rev Public Health.

[5] Cornelius ME, Wang TW, Jamal A, Loretan CG, Neff LJ (2020). Tobacco product use among adults—United States, 2019. Morb Mortal Wkly Rep.

[6] Frieden TR (2010). A framework for public health action: the health impact pyramid. Am J Public Health.

[7] Giebel GD, Speckemeier C, Abels C, Plescher F, Börchers K, Wasem J, Neusser S (2023). Problems and barriers related to the use of digital health applications: scoping review. J Med Internet Res.

[8] Gültzow T, Neter E, Zimmermann HML. Making research look like the world looks: introducing the ‘Inclusivity & Diversity Add-On for Preregistration Forms’ developed during a EHPS2022 pre-conference workshop. Eur Health Psychol. 2023;22(6):1063-9.

[9] Harzing AW (2006). Response styles in cross-national survey research: a 26-country study. Int J Cross Cult Manag.

[10] Hyman A, Stewart K, Jamin AM, Lauscher HN, Stacy E, Kasten G, Ho K (2020). Testing a school-based program to promote digital health literacy and healthy lifestyle behaviours in intermediate elementary students: the Learning for Life program. Prev Med Rep.

[11] International Telecommunication Union (2021). Measuring digital development. Facts and figures 2021. Retrieved from: https://www.itu.int/en/ITU-D/Statistics/Documents/facts/FactsFigures2021.pdf.

[12] Jahnel T, Dassow HH, Gerhardus A, Schüz B (2022). The digital rainbow: digital determinants of health inequities. Digital Health.

[13] König LM, Attig C, Franke T, Renner B (2021). Barriers to and facilitators for using nutrition apps: systematic review and conceptual framework. JMIR Mhealth Uhealth.

[14] Korbmacher M, Azevedo F, Pennington C, Hartmann H, Pownall M, Schmidt K, Evans T (2023). The replication crisis has led to positive structural, procedural, and community changes. Nat Commun Psychol..

[15] Kwasnicka D, Keller J, Perski O, Potthoff S, ten Hoor GA, Ainsworth B, König LM, Sanderman R (2022). White Paper: Open Digital Health – accelerating transparent and scalable health promotion and treatment. Health Psychol Rev.

[16] Lyles CR, Nguyen OK, Khoong EC, Aguilera A, Sarkar U (2023). Multilevel determinants of digital health equity: a literature synthesis to advance the field. Annu Rev Public Health.

[17] Marmot, M. Health inequalities in the EU. Final report of a consortium. European Commission Directorate-General for Health and Consumers. 2023. https://ec.europa.eu/health//sites/health/fles/social_determinants/docs/healthinequalitiesineu_2013_en.pdf.

[18] McPhail A, Hare ME, Talcott GW, Little MA, Bursac Z, Krukowski RA (2023). Gestational weight gain during the COVID-19 pandemic. Matern Child Health J..

[19] Müller AC, Wachtler B, Lampert T (2020). Digital divide-soziale unterschiede in der nutzung digitaler gesundheitsangebote. Bundesgesundheitsblatt Gesundheitsforschung Gesundheitsschutz.

[20] Office for National Statistics. Exploring the UK’s digital divide. 2019. Retrieved from: https://www.ons.gov.uk/peoplepopulationandcommunity/householdcharacteristics/homeinternetandsocialmediausage/articles/exploringtheuksdigitaldivide/2019-03-04.

[21] O’Neill J, Tabish H, Welch V, Petticrew M, Pottie K, Clarke M, Tugwell P (2014). Applying an equity lens to interventions: using PROGRESS ensures consideration of socially stratifying factors to illuminate inequities in health. J Clin Epidemiol.

[22] Pérez-Muñoz A, Horn TL, Graber J, Chowdhury SMR, Bursac Z, Krukowski RA (2022). Recruitment strategies for a post cessation weight management trial: a comparison of strategy cost-effectiveness and sample diversity. Contemp Clin Trials Commun.

[23] Pew Research Center. Mobile Fact Sheet. 2021. Retrieved from: https://www.pewresearch.org/internet/fact-sheet/mobile/.

[24] Pratap A, Neto EC, Snyder P, Stepnowsky C, Elhadad N, Grant D, Omberg L (2020). Indicators of retention in remote digital health studies: a cross-study evaluation of 100,000 participants. NPJ Digital Med.

[25] Sauermann S, Herzberg J, Burkert S, Habetha S (2022). DiGA–a chance for the German healthcare system. J Eur CME.

[26] Scholes, S., & Neave, A. Health survey for England 2016: physical activity in adults. leeds: health and social care information centre. 2017. Retrieved from: https://data.londonsport.org/download/23q8d/2x8/LSR332%20NHS%20Digital%20-%20Health%20Survey%20for%20England16%20-%20Adult%20physical%20activity.pdf.

[27] Szinay S, Forbes CC, Busse H, DeSmet A, Smit ES, König LM. Is the uptake, engagement and efficacy of exclusively mobile interventions for the promotion of weight-related behaviours equal for all? A systematic review. Obes Rev. 2023;24(3):e13542. 10.1111/obr.13542 36625062

[28] U.S. Food and Drug Administration. What is Digital Health? 2020. Retrieved from: https://www.fda.gov/medical-devices/digital-health-center-excellence/what-digital-health.

[29] van der Ham M, Bolijn R, de Vries A, Ponce MC, van Valkengoed IG (2021). Gender inequality and the double burden of disease in low-income and middle-income countries: an ecological study. BMJ Open.

[30] Western MJ, Armstrong ME, Islam I, Morgan K, Jones UF, Kelson MJ (2021). The effectiveness of digital interventions for increasing physical activity in individuals of low socioeconomic status: a systematic review and meta-analysis. Int J Behav Nutr Phys Act.

[31] Whitehead M, Dahlgren G (1991). What can be done about inequalities in health?. The Lancet..

